# Feasibility study for interactive reporting of network meta-analysis: experiences from the development of the MetaInsight COVID-19 app for stakeholder exploration, re-analysis and sensitivity analysis from living systematic reviews

**DOI:** 10.1186/s12874-022-01507-x

**Published:** 2022-01-22

**Authors:** Yiqiao Xin, Clareece R. Nevill, Janion Nevill, Ewan Gray, Nicola J. Cooper, Naomi Bradbury, Alex J. Sutton

**Affiliations:** 1 NIHR Complex Review Support Unit, Health Technology Assessment and Health Economics (HEHTA), Institute of Health and Wellbeing, University of Glasgow, Glasgow, UK; 2 NIHR Complex Review Support Unit, Department of Health Sciences, Centre for Medicine, University of Leicester, University Road, Leicester, LE1 7RH UK; 3 Independent researcher, Tewkesbury, UK; 4 Health Economist, Freelance Health Economics consultant, East Lothian, UK; 5 Zeeman Institute: Systems Biology and Infectious Disease Epidemiology Research (SBIDER), School of Life Sciences, University of Warwick, Coventry, UK

**Keywords:** Network meta-analysis, Living systematic review, Covid-19

## Abstract

**Background:**

Network meta-analysis (NMA) has been increasingly adopted worldwide by Cochrane reviews, guideline developers and decision-making bodies to identify optimal treatment choices. However, NMA results are often produced statically, not allowing stakeholders to ‘dig deeper’ and interrogate with their own judgement. Additionally, amid the COVID-19 pandemic, unnecessary or duplicated reviews have been proposed which analyse from the same pool of evidence. We developed the ‘MetaInsight COVID-19’ app as a prototype for an interactive platform to eliminate such duplicated efforts, by empowering users to freely analyse the data and improve scientific transparency.

**Methods:**

MetaInsight COVID-19 (https://crsu.shinyapps.io/metainsightcovid/) was developed to conduct NMA with the evolving evidence on treatments for COVID-19. It was updated weekly between 19th May – 19th Oct 2020, incorporating new evidence identified from a living systematic review.

**Results:**

The app includes embedded functions to facilitate study selection based on study characteristics, and displays the synthesised results in real time. It allows both frequentist and Bayesian NMA to be conducted as well as consistency and heterogeneity assessments. A demonstration of the app is provided and experiences of building such a platform are discussed.

**Conclusions:**

MetaInsight COVID-19 allows users to take control of the evidence synthesis using the analytic approach they deem appropriate to ascertain how robust findings are to alternative analysis strategies and study inclusion criteria. It is hoped that this app will help avoid many of the duplicated efforts when reviewing and synthesising the COVID-19 evidence, and, in addition, establish the desirability of an open platform format such as this for interactive data interrogation, visualisation, and reporting for any traditional or ‘living’ NMA.

**Supplementary Information:**

The online version contains supplementary material available at 10.1186/s12874-022-01507-x.

## Background

Traditional meta-analysis, or ‘pairwise meta-analysis’, is used to compare two interventions or treatment options. It is therefore limited in its ability to answer clinically relevant questions, such as the ‘most’ effective intervention where three or more are involved. Network meta-analysis (NMA) is the standard method for synthesising quantitative evidence when more than two interventions are compared across multiple studies [1–4]. As such, NMA has been increasingly adopted worldwide in recent years by Cochrane reviews, guideline developers and decision-making bodies to identify the optimal treatment choices for a given indication. Despite the popularity, the results of NMA have been traditionally produced statically, precluding stakeholders to interrogate the evidence with their own judgements and preferences.

As of 19th October 2020, the severe acute respiratory syndrome coronavirus 2 (SARS-CoV-2) has caused more than 40.1 million confirmed cases of coronavirus disease 2019 (COVID-19) worldwide, resulting in 1.1 million deaths and enormous impact on many aspects of living for individuals and society [5]. Impacts from COVID-19 include, but are not limited to, a significant increased burden on healthcare systems, disruption within the economy, and disturbance of social activities including religious and cultural events [6]. Since the first trial on COVID-19 was published in April 2020, the evidence surrounding treatments for COVID-19 has been evolving rapidly; as of 5th October 2020, 2388 clinical trials worldwide for treating COVID-19 have been registered [7]. It has been recognised that extensive research needs to be undertaken on many areas on COVID-19, including medication/therapy [8]. A great variety of treatment options are being tested simultaneously, covering pharmacological therapies such as hydroxychloroquine and azithromycin which have claimed to be effective [8], biological therapies such as plasma-based therapy and immunoglobulins, devices such as non-invasive respiratory support and traditional therapies such as Chinese traditional medicine [7]. Consequently, an increasing number of protocols from researchers and clinicians worldwide have been registered, aiming to synthesise evidence from randomised trials and/or observational studies of one or more interventions, investigating outcomes such as efficacy and safety for COVID-19 patients of graded severity. As of 19th October 2020, 338 systematic review (SR) protocols of treatment for COVID-19 have been registered in the international SR registry, PROSPERO [9].

Exacerbated by the urgency of COVID-19 research, there is potential for huge amounts of duplicated or unnecessary work being created from conducting SRs containing syntheses using the same pool of data. The difference between most of these protocols lies in drawing varied subgroups to suit specific research questions. We believe many of these redundant and time-consuming efforts can be avoided through establishing an interactive web-based platform to conduct NMA. The platform would allow exploration of the up-to-date evidence, detailed subgroup analysis, and re-analysis with different models as deemed appropriate by the different stakeholders. Previously we developed MetaInsight, a freely available user-friendly web app for conducting NMA [10]. In response to the coronavirus pandemic, we have developed a specially tailored version of MetaInsight, built around up-to-date trial evidence (up to 19th October 2020) on pharmacological treatments for COVID-19, namely MetaInsight COVID-19 [11]. It was developed to allow exploration, re-analysis, sensitivity analysis, and interrogation of data from existing living systematic reviews (LSRs) of treatment for COVID-19.

The aim of this feasibility study was to show that a tool such as MetaInsight COVID-19 would add value to a LSR, through illustrating how features of such a tool benefit exploration and communication of results. We also discuss the generalisability of developing an online interactive analysis and reporting app for any specific living or non-living NMA.

## Methods

### MetaInsight

Traditionally NMA is conducted with statistical software such as WinBUGS, OpenBUGS, STATA or R, which can form a barrier for people who are inexperienced with such software but have knowledge of NMA. MetaInsight provides a user-friendly “point and click” interface for NMA and thus makes such analyses more accessible and efficient for the research community. MetaInsight requires only a web browser but leverages established analysis routines in R [10]. It allows users to upload their own datasets and provides interactive graphical representations of the treatment network and various aspects of the NMA results including treatment ranking. MetaInsight has been built using R Shiny [12], which allows users to build interactive web applications and host them on a server. The NMA analysis is conducted using the R packages netmeta [13] and GeMTC [14].

The latest version of MetaInsight, released in April 2020 [15], includes both frequentist and Bayesian NMA functionalities as well as further functionality enhancements compared to previous versions. Since its inception, MetaInsight has been actively evolving with new features being developed to improve users’ experience. These include network disconnection notifications, assessment of model fit, interactive plots for checking inconsistency, a comprehensive step-by-step user guide [16], and a trouble-shooting page (addressing the common errors users experience). Currently the app is used worldwide for approximately 1000 h per month.

### Data source

In contrast to MetaInsight, where users analyse their own data, MetaInsight COVID-19 is specifically tailored around the evolving evidence from randomised controlled trials on the effectiveness of pharmacological treatments for COVID-19. We anticipate future research groups to run the review of literature and publish/produce the meta-analysis together; however, the aim of this feasibility case study does not include defining or exploring selection criteria regarding the systematic review itself. Therefore, data from an external review group was purely used to move the feasibility case study forward. Several groups around the world are currently carrying out living mapping and/or LSRs on this topic and frequently update the evidence on their websites (The COVID-NMA initiative [17], EPPI Center [18], and The LIVING Project [19]). We included data from ‘The COVID-NMA initiative’ which is conducted and supported by a broad international consortium led by Cochrane France. They were selected for this feasibility case study for their efficient and rigorous approach to identifying relevant studies, quick establishment, clear presentation of findings, and public availability of the data on their website. Furthermore, they continually updated and critically appraised the evidence as new studies became available. Data was extracted from the SR itself using a script written in Python (details in the results section), rather than following up the individual studies included by the COVID-NMA initiative. As the focus of our study was on creating the analysis tool itself, we prioritised developing the tool rather than validating the SR.

### Data analysis

MetaInsight COVID-19 has all the functionality that MetaInsight has, which includes a vast range of analyses available for the user to carry out network meta-analyses. To give the app some focus and direction, a front summary page was included to the MetaInsight COVID-19 prototype. The elements of the front summary page revolved around a single network meta-analysis using a random-effects model with frequentist methodology; the results were presented using a forest plot.

At the time of data extraction, instead of carrying out network meta-analyses, the COVID-NMA initiative were only carrying out pairwise meta-analyses, with subgroup analyses for disease severity.

## Results

### Retrieval of data

The COVID-NMA initiative website was monitored during 19th May – 19th October 2020; any new evidence was added to our app weekly through a semi-automated process. A script was written in Python to detect any changes in the forest plots across the website (these were the only point of access to extract the necessary data from the website). It downloaded all the forest plots and compared the pixels against those from images downloaded previously. The script highlighted areas of change on the respective images and emailed them to a team member. It also detected if any new forest plots were added (i.e. new treatment comparisons). The Python script was loaded onto a web-connected Raspberry Pi (a small PC) programmed to run the script every week. The team member receiving the emails then manually added to or amended the cloud-based datasheet that was connected to the app; thus keeping the evidence-base up-to-date. This process was unique to the project; we do not anticipate that future research groups would have to use similar methods, as one would hope that either the review and meta-analysis groups are as one, or that direct access to data would be available.

### Output and features of MetaInsight COVID-19

Three binary outcomes of interest were included in this prototype based on their importance to clinical decision-making and frequency of being measured in the trials: all-cause mortality; incidence of viral negative conversion; and serious adverse events. The user can switch between outcomes by selecting the respective radio button choices.

As well as the forest plot of the resulting random-effects model, other features were included on the summary front page. To facilitate the comparison of characteristics between studies, comprehensive study characteristics and outcome data were extracted and tabulated for each outcome. These included: author, treatments, number of people who had the outcome in each arm, sample size in each arm, follow-up days, dose, treatment duration, risk of bias assessment, severity of COVID-19, country, and time of outcome measure (days). The characteristics table doubled-up as functionality to exclude any studies from the synthesis, allowing users to explore how the analysis changes. Furthermore, a network plot was available to summarise the treatments in the synthesis and evaluate their connectivity.

By including all the options available in the main MetaInsight app, users were also able to alter the analysis in the following ways to enable interrogation of the review further: use a fixed-effects model; use Bayesian methodology; access further summary plots; access simultaneous comparisons of results using different selection of studies to aid sensitivity analysis.

## Demonstration

A demonstration of MetaInsight COVID-19 can be found in Additional file 1.

## Discussion

The future of evidence synthesis should be living and open; ‘living’ refers to continuously updated searching, extraction, appraisal, and analysis of evidence [20] whereas ‘open’ refers to offering the end users freedom to re-analyse and interrogate the data and is consistent with the aims of open science [21, 22] more generally. In this way, MetaInsight COVID-19 promotes the proposal of ‘open synthesis’ where the open science principle is applied to the full process of evidence synthesis, including freely accessible detailed open methods, data, and repeatable programmatic code [21, 22]. More specifically, MetaInsight addresses the practicality of open coding and open data; in many situations, capacity is lacking for clinicians, or other non-statistical expert stakeholders, to run others’ code in specific statistical software and re-analyse the downloaded data. With its easy-to-use interface, MetaInsight provides a shortcut to stakeholders for assessing the uncertainty of the NMA result by innovating the process of data interrogation with a point-and-click interface, making meta-analysis results instantly open to critique and interrogation.

MetaInsight COVID-19 was developed as a motivating test case for a specific interactive NMA tool for use with ‘living’ evidence. It aims to provide stakeholders with the ability to quickly explore and interrogate the up-to-date available trial evidence and conduct detailed analyses tailored to their own needs and preferences rather than relying on specific static published reviews. This study therefore illustrates a successful ‘proof of concept’ that an interactive explorable, critiquable and customisable (network) meta-analysis can be easily updated as evidence emerges, fitting both the static and living systematic review frameworks. All app features are user-friendly by utilising interactive point-and-click tools. The utilisation of such beneficial features include: i) Limiting the evidence base to trials with certain characteristics, such as trials concerning patients with severe COVID-19 or trials of a certain sample size (this also applies to trials not connected to the initial primary network); ii) Assessing the impact of statistical model choice on conclusions (e.g. fixed-effects vs random-effects, frequentist vs Bayesian methods); iii) Assessing the impact of individual trials on overall model fit or network inconsistency, and the impact of excluding specific trials from the analysis on the results facilitated by presenting results side-by-side; iv) Conducting pairwise meta-analysis on a specific treatment contrast(s) by using simple filtering functions within the ‘pop-up’ data table; and v) Simultaneously investigating any combination of the above. The availability of such features is the cornerstone of the app being such an advantageous tool for users to explore the up-to-date evidence effectively themselves. Furthermore, more experience users have the option of adding more trials to the evidence-base that is used by MetaInsight COVID-19. Users need only add new trials to the datasheet and the app with automatically re-run all of the analyses. If tailored and used by an active research team conducting a living review, they would highly benefit from the aforementioned advantage of the analysis automatically updating, allowing the team to work more efficiently.

We recognise that there are similar studies in the recent literature, but find that the primary focus varies. PsychOpen CAMA is a platform for open and cumulative meta-analyses in psychology, developed by the Leibniz Institute for Psychology [23], MetaLab is a set of interactive, community-augmented meta-analysis tools for cognitive development research [24], and Ahern et al. [25] developed an interactive web-app for the meta-analysis of CYP2D6 impairment and tamoxifen failure. Whilst all four applications have a friendly graphical user interface with meta-analytic functionalities and graphical outputs openly available, PyschOpen CAMA and MetaLab have a focus on community-augmented meta-analysis, which encourages the research community to be involved with the provision of data. However, our feasibility study did not set out to investigate the data collection aspect of living NMAs. MetaLab has further functionality including power analysis and simulation, and MetaInsight COVID-19 has sensitivity analysis functionality. All four applications have a repository from which users can analyse a specified subset, however, PyschOpen CAMA has a much wider subject area and functionality for studies to be selected automatically based on user selected topics and questions, rather than study characteristics as in MetaInsight COVID. The app developed by Ahern et al. is very similar to MetaInsight COVID, particularly as both were developed using the same software. However, MetaInsight COVID has wider functionality and a summary front page. Compared to the websites sharing results from living meta-analyses of COVID-19 treatments, MetaInsight COVID-19 has the advantage of allowing the user to make changes to the analysis model and selection of studies as they see appropriate.

Other non-living generic interactive web-applications exist within the field of evidence synthesis, including CINeMA [26] and L.OVE [27]. Whilst the L.OVE platform is expansive, the data is accumulated via a network of experts and algorithms, therefore it does not allow users to analyse their own data, or make changes (e.g. excluding studies or changing analysis model) to the loaded systematic reviews. CINeMA is a useful tool focusing on the important issues of bias, uncertainty, and checking of assumptions, and can be used alongside MetaInsight. To aid comparison, the default NMA presented in MetaInsight COVID-19 was run using CINeMA (results in additional file 2). It is important to note, that whilst both apps conduct NMA, CINeMA’s focus is not on presenting the summary results of the analysis. CINeMA had further design options regarding the network plot (additional file 2 – Fig. 1) and gave a useful, visually appealing table of the Risk of Bias contributions (additional file 2 – Fig. 2). However, results were limited as it needed extra data on indirectness and clinical expertise to define certain conditions needed to evaluate incoherence etc. (further details in additional file 2). We consider MetaInsight to be a more intuitive tool for novice users, immediately giving visual summaries of the NMA results and facilitating sensitivity analysis.

The extent to which the NMA results are valid is contingent on the appropriateness of combining the different studies together, the decisions required to do so inevitably have a degree of subjectivity. This subjectivity may be aggravated with the increasing number of LSRs leading to limited numbers of authorship groups; different authors may reach different conclusions with the same evidence [28, 29]. MetaInsight empowers all stakeholders to explore the robustness of results by freely varying the data inclusion criteria and analysis assumptions. In the COVID-19 situation, the degree of heterogeneity of the studies is large, possibly due to the nature of an emergency. The published and ongoing trials differ in various aspects such as definition of standard care, population characteristics, outcome measures and their definitions, duration and dosage of the interventions, and perhaps most importantly their quality/risk of bias. As others have noted [25], traditional NMA provides static evidence summaries which do not allow stakeholders to fully explore the analysis themselves, some of whom may have different views on key features of the analysis. Through tools such our app, users can, with ease, take control of the evidence synthesis using their preferred analytic approach. Such a tool would also complement emerging technologies such as the Internet of (Medical) Things (an interconnected network of medical devices and/or operations) facilitating transparency of COVID-19 treatments to patients [30, 31]. The capability of fine-tuning analysis results within LSR apps would also go hand-in-hand with Industry 5.0-based technologies to help provide more personalised therapy and treatments; a potential benefit for the COVID-19 pandemic [32].

MetaInsight COVID-19 was developed rapidly leveraging our existing MetaInsight app [10]. Practically, we also found it beneficial to use an online shared document to store the extracted data which was used to load data into the app. It allowed multiple team members to access and edit the data, and included version control/history. It also saves the hassle of re-publishing the entire code base when data were changed or new data were added.

MetaInsight is designed for health researchers without specific programming expertise with respect to carrying out state-of-the-science analyses such as NMA, as well as experts seeking convenient and efficient solutions or needing a practical teaching tool in the classroom. We have observed an increased usage of our app and queries by researchers in many areas of the world, including those in low- and middle-income countries, which demonstrates the demand and its value. In addition to its primary function of allowing non-specialists do research using NMA, we get enthusiastic reports from statisticians who use it because of its ease and efficiency and educators who use it due to its visualisation features.

Challenges were met during the development of MetaInsight COVID-19, many were overcome but some were not resolved. We believe that briefly outlining these unresolved challenges will be informative for similar initiatives in the future. Interventions with different doses/durations within a trial were recorded as separate nodes (i.e. treatments) within the network (e.g. Remdesivir 5 days and 10 days). Node merging (e.g. combining the 5 and 10 day durations) and splitting would allow more flexibility within analyses, letting users group and separate interventions as they need. The trials mostly compared against standard care or placebo. We treated them as different interventions whereas others may decide they could be combined (e.g., as seen in the COVID-NMA initiative [17]). In situations like this, node merging/splitting would provide users more freedom. In addition, further investigation should be conducted on the definition of standard care and placebo group. From our experience, initially, all versions of standard care were treated equally within the network, however, concerns were raised when standard care in later trials included interventions already within the network. Researchers should be aware of this issue within LSRs (especially in fast-paced areas such as COVID-19).

### Limitations

This app is limited to NMAs using aggregated (summary) data from studies. NMA with individual patient data can facilitate standardisation of analyses across studies such as standardising the inclusion/exclusion criteria, statistical analysis approaches, adjustment of baseline factors, and accounting for correlation between multiple end points etc. [33] We are also aware that separate users of the app cannot interact directly or discuss results, insights, or raise questions. The inclusion of a feedback discussion board is a further possible enhancement.

Whilst the app currently allows the user to assess the impact of statistical model choice and individual trials, it does not allow inclusion of factors such as Risk of Bias or GRADE assessments of the evidence. Integrating this information could be a worthwhile extension, and we have ideas for moving this forward. As implemented by others, there could be functionality to allow the user to explore the effect of down-weighting evidence due to quality concerns in the synthesis [25]. Secondly, something similar to the functionality developed by the authors in an app for meta-analysis of diagnostic test accuracy could be explored, which implemented visualisation of Risk of Bias to inform sensitivity analysis [34].

A largely unresolved challenge for LSRs is updating statistical analyses while addressing the issue that the type-1 error rate will increase with each update (i.e. a statistically significant treatment effect will be detected when one does not really exist) [35]. Trial sequential analysis [36] has been proposed as a solution to this problem, but whilst its application to NMA has started to be developed [37] along with respective R packages [38], there are still issues that need further work such as futility boundaries. We stress this is a general issue with LSRs containing NMA and not a specific limitation of the app, but an important issue for the user to bear in mind nonetheless.

### Future directions

Going forward, a series of functions are in the pipeline for the next version of MetaInsight, including adding covariates to enable meta-regression, 3D plots to visualise treatment effects and uncertainty, enabling survival outcomes in the analysis, and more customisability of analysis details, etc. Most of these function needs were identified through our training events or technical support with users, demonstrating their use for stakeholders and in the long term contribute to improved healthcare decision making. We hope to make such functionality available in our COVID-19 tailored app also.

We recognise that applications such as this are powerful and may give misleading impressions regarding the ease of conducting analyses such as NMA; therefore, they should be used with caution. Whilst MetaInsight COVID-19 contains a generic message of caution, encouraging users to ensure that they have suitable statistical support for their project, future work on MetaInsight and variations would include specific advisory pointers to the end-user. For example, (i) advising users to consider factors such as the population of interest, outcome definitions, and intervention doses etc. when comparing their personalised analysis to the original, (ii) warnings of potential bias from continuity corrections being applied when included studies have reported zero outcomes, and (iii) guidance surrounding the effect uninformative priors may have on Bayesian analyses with little data.

A potential future direction that was not part of our aim of this study, but is appealing, is a version of MetaInsight that would allow exploration of trials across different existing SRs (through switching between analysis models, studies included, data used etc. based on existing reviews). This could help reconcile any differences in findings observed in different SRs.

A further challenge we are researching is developing a way of making an available template so others can create a similar app for their specific LSR(s), allowing readers similar freedom and functionality. Even static and traditionally published meta-analyses could benefit from making their data available in such an app to improve transparency and enable readers to “dig deeper” into the analysis.

## Conclusions

MetaInsight COVID-19 allows users to take control of the evidence synthesis using the analytic approach they deem appropriate to ascertain how robust findings are to alternative analysis strategies and study inclusion criteria. It is hoped that this app will help avoid many of the duplicated efforts when reviewing and synthesising the COVID-19 evidence, and, in addition, establish the desirability of an open platform format such as this for interactive data interrogation, visualisation, and reporting for any traditional or ‘living’ NMA.

## Supplementary Information

**Additional file 1.** Demonstration of MetaInsight COVID19.docx - Demonstration of MetaInsight COVID-19 App – A detailed demonstration of how to run and use MetaInsight COVID-19.

**Additional file 2.** NMA conducted with CINeMA.docx – NMA conducted with CINeMA – A report of conducting the same NMA using the online web tool CINeMA.

## Data Availability

Data sharing is not applicable to this article.

## Abbreviations

NMA: Network Meta-analysis
SARS-CoV-2: Severe acute respiratory syndrome coronavirus 2
COVID-19: Coronavirus disease 2019
SR: Systematic review
LSR: Living systematic review
