# Supplementary material for: Feasibility study for interactive reporting of network meta-analysis: experiences from the development of the MetaInsight COVID-19 app for stakeholder exploration, re-analysis and sensitivity analysis from living systematic reviews
Source: BMC Med Res Methodol. 2022 Jan 22;22:26. doi: 10.1186/s12874-022-01507-x (PMC8783587; doi:10.1186/s12874-022-01507-x)
Supplement: Supplementary file 1 — Additional file 1. Demonstration of MetaInsight COVID19.docx - Demonstration of MetaInsight COVID-19 App – A detailed demonstration of how to run and use MetaInsight COVID-19. [file 12874_2022_1507_MOESM1_ESM.docx]

**Supplemental Material: DEMONSTRATION OF METAINSIGHT COVID-19 APP**

**Title:** Feasibility study for interactive reporting of network meta-analysis: Experiences from the development of the MetaInsight COVID-19 app for stakeholder exploration, re-analysis and sensitivity analysis from living systematic reviews

**Authors:** Yiqiao Xin^1*^, Clareece R Nevill^2*^, Janion Nevill^3^, Ewan Gray^4^, Nicola J Cooper^2^, Naomi Bradbury^5^, Alex J Sutton^2^.

^*^Joint first authorship

**Affiliations:**

1. NIHR Complex Review Support Unit, Health Technology Assessment and Health Economics (HEHTA), Institute of Health and Wellbeing, University of Glasgow, UK.
2. NIHR Complex Review Support Unit, Department of Health Sciences, University of Leicester, UK.
3. Independent researcher, Tewkesbury, UK.
4. Health Economist, Freelance Health Economics consultant, East Lothian, UK.
5. Zeeman Institute: Systems Biology and Infectious Disease Epidemiology Research (SBIDER), School of Life Sciences, University of Warwick, Coventry, UK.

**Corresponding author contact address:**

Alex J Sutton, Department of Health Sciences, Centre for Medicine, University of Leicester, University Road, Leicester, LE1 7RH. [ajs22@leicester.ac.uk](mailto:ajs22@leicester.ac.uk)

## The MetaInsight COVID-19 app (<https://crsu.shinyapps.io/metainsightcovid/>) contains five tabs. The front tab, titled ‘COVID19’, includes a date indicating when the data was last checked. This initial tab provides a ‘top-level’ summary of the NMA results for the selected outcome. It displays the study characteristics and outcome table, the network plot with two choices of style, and summary results shown as a forest plot. The page includes embedded functions to facilitate users to freely select studies to combine, as deemed appropriate based on the characteristics table, and the results are displayed in real time. The next tab, ‘Project introduction’, provides general information on background, data source, team members and contact information. The third tab, titled ‘Detailed data analysis’, contains the regular MetaInsight app’s various functionalities including allowing the users to choose between fixed effect and random effect models, select the outcome measures, conduct frequentist or Bayesian analysis, and check the treatment ranking. This also includes facilities for advanced usage including checking of modelling assumptions via cross validation, the use of model fit statistics to choose between competing statistical models and use of the residual deviance plots to examine the model fit and detect outlying results utilising Bayesian simulation methods calculated calling the external program JAGS [Plummer, M. (2003). JAGS: A program for analysis of Bayesian graphical models using Gibbs sampling]. The app also allows the user to download the R code used 'under the hood' that carried out the analysis to ensure reproducibility of research. On both the front ‘COVID19’ and the ‘detailed data analysis’ tab, subgroup analysis can be undertaken by selecting specific studies which can be filtered and excluded from the analysis, the analysis model changed, and assessments of network coherence carried out. We hope much of the functionality is self-explanatory, but a full manual is available on the ‘User Guide’ tab. Finally, known issues as they are discovered are listed on the final tab before they are resolved.

## Illustrative example of how the MetaInsight COVID-19 app can be used

The illustrative dataset, available in the app, includes all the randomised clinical trial evidence of pharmacological treatments for COVID-19 for all-cause mortality available on the covid-nma.com website up to 19th October 2020 (the finish date of this pilot).

## Outcome selection

As mentioned above, users can select from the three outcomes of interest to load data into the app (Figure 1). The corresponding data will show in real time in the data table underneath. For this example the outcome all-cause mortality is selected.


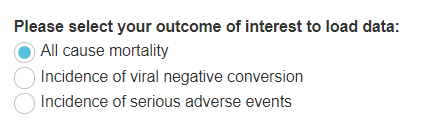


Figure 1 Screenshot of selection of outcome of interest

## Characteristics table

The table shows the characteristics of the included studies (Figure 2). Within this example, the trial interventions do not form a cohesive (i.e. connected) network of evidence e.g. chloroquine 600mg and chloroquine 450mg are not trialled with any other interventions in the network. Consequently, more than one network exists. To address this, we created a column titled ‘Included’ to indicate which studies are currently connected to the primary network (unconnected studies were labelled ‘not yet’). In the default option, studies which are not yet connected are not included in the NMA.


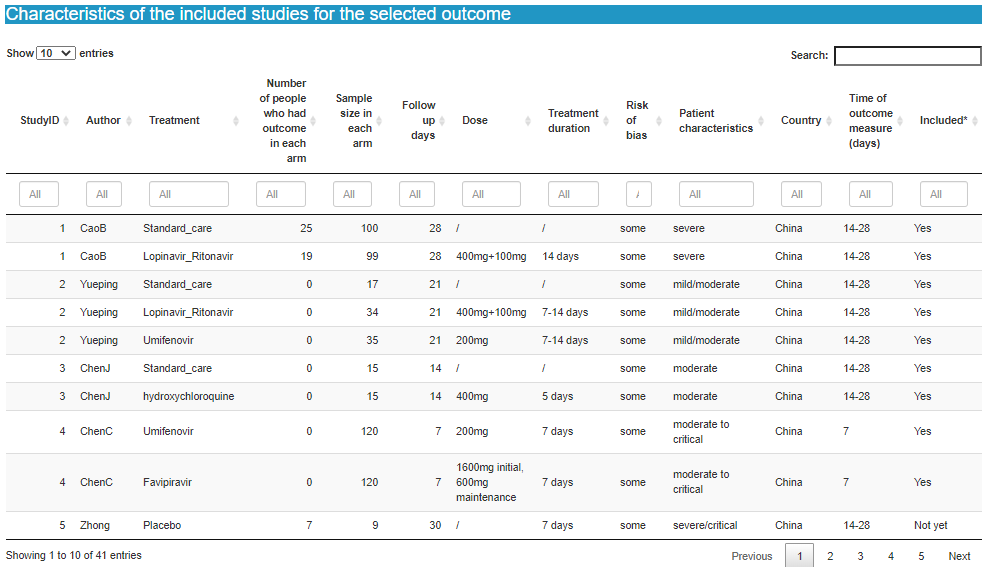


Figure 2 Screenshot of the characteristics table of the included studies for the all-cause mortality outcome

## Fully interactive selection of studies to include in the analysis

The app provides full flexibility regarding study selection which means that users have the freedom to select any study into the analysis, providing they together form a connected network. This can be easily done by manual study selection, either through individually clicking upon the study, or using the filter function.

For example, if users are interested in the direct evidence associated with Remdesivir, an analysis of the small network of trials which evaluate Remdesivir may be of interest. To investigate this Remdesivir centred network, users can firstly use the filter option to identify the studies that assessed Remdesivir as shown in Figure 3; in this case it is studies 6, 9, 10 and 33. Then all the studies can be selected by removing all the filters, i.e. removing the treatments in the filter box. This is necessary because the filtering only picks up the *individual* *arms* that contain Remdesivir, whilst the desired analysis requires *all of the arms* of studies that contain Remdesivir in one (or more) arm. Now, moving back to the all study view we are able to select all the arms from the four relevant studies (i.e. studies 6, 9, 10 and 33). The ‘View the included studies after filtering or individual selection (click to open / hide this panel)’ button can be pressed to view the selected studies to double check the current inclusion is correct (Figure 4).


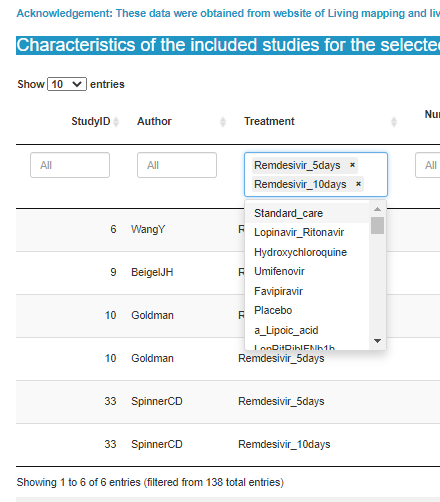


Figure 3 Screenshot of the filter option used to select only the studies assessing Remdesivir


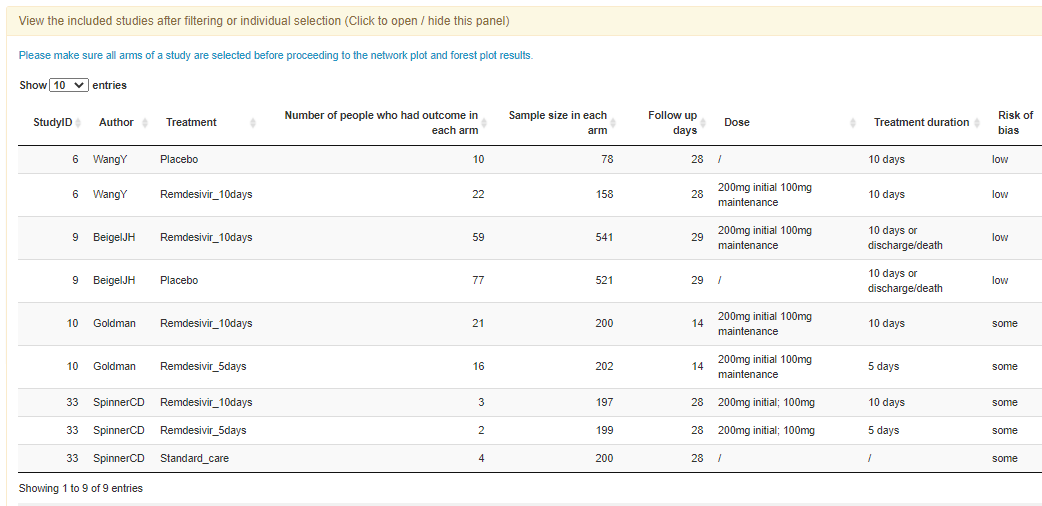


Figure 4 Screenshot of the study inclusion checking when studies that evaluate Remdesivir (numbers 6, 9, 10 and 33) are selected

## Network plot

Underneath the table is the network plot of the selected studies. By selecting the radio buttons users can choose between two visualisation styles, as shown in Figure 5 A & B. Style A has additional criteria for inclusion of data. When both arms (of a 2-arm trial) have zero events, which means the treatment effect is not defined, the study will not be analysed and will not show in this network plot. Since many of the Covid-19 trials have zero deaths for both arms this can have considerable impact on the appearance of the network for ‘all-cause mortality’ compared to Style B which includes such studies. Similarly, when a multi-arm study is included and only one of its arms has an outcome not equal to zero, the style A network will not include it. These warnings are highlighted underneath the plot when style A is selected. Style B is not affected by the outcome data reported but solely reflects the study design.


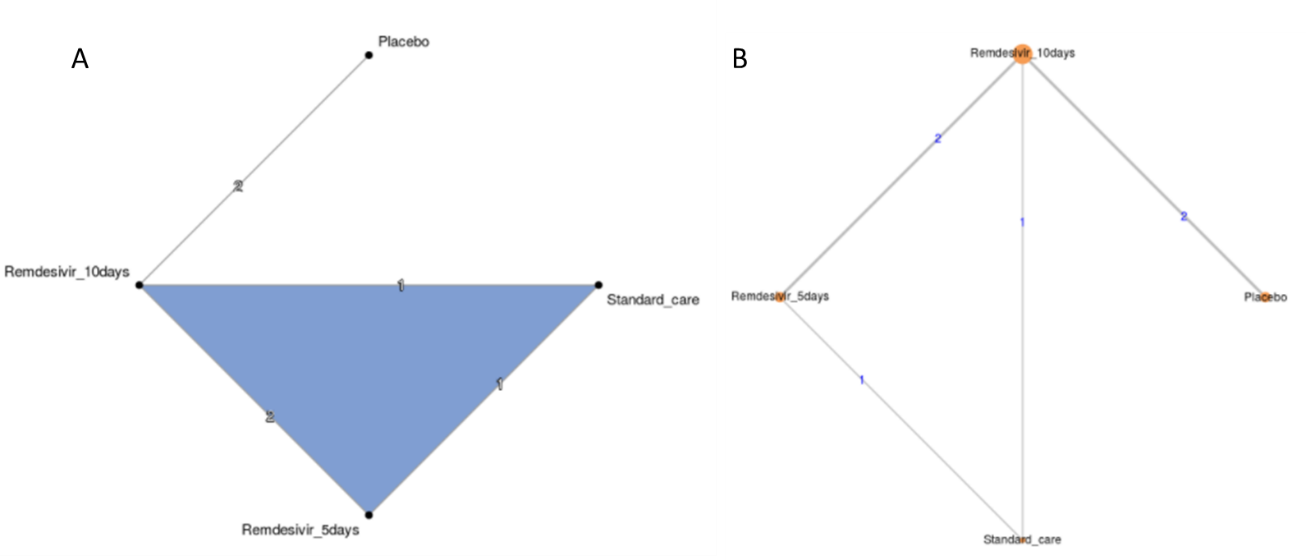


Figure 5 Screenshot of two styles of network plot: A. number of trials shown on the line, shaded areas indicating multi-arm trials; B. number of people indicated by size of node and number of trials shown on the line.

## Forest plot

Forest plots provide the resulting pooled effect estimates from an NMA and their 95% confidence intervals, for all interventions compared with the reference treatment. In this app, we used ‘standard care’ as reference treatment if the network includes standard care. When standard care is not included, the app will search if ‘placebo’ is included in the network. If yes, placebo will be used as reference treatment; and if not, the reference treatment will be randomly selected from the included treatments in the network. On the front page, we present the forest plot following frequentist analysis using the ‘netmeta’ R package, with the random effects model here assuming there exists between-study variation due to the heterogeneity between trials (Figure 6). The result for the Remdesivir studies showed that for the outcome of all-cause mortality, Remdesivir on a 5-day regimen may be more effective than Remdesivir on a 10-day regimen, and both may be more effective than standard care. However both comparisons have limited evidence and the resulting confidence intervals are wide. Neither comparison showed a difference which achieved statistical significance at conventional (P<0.05) levels. If all the filters are removed so all studies are considered (by deselecting the rows of study 6, 9, 10 and 33), the result of all studies connected in the primary network that do not have a missing outcome will be shown (Figure 7).


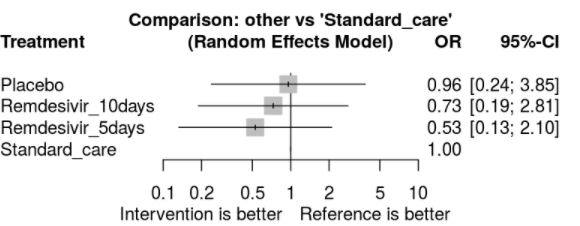


Figure 6 Screenshot of forest plot result of Remdesivir studies using frequentist analysis with a random effects model


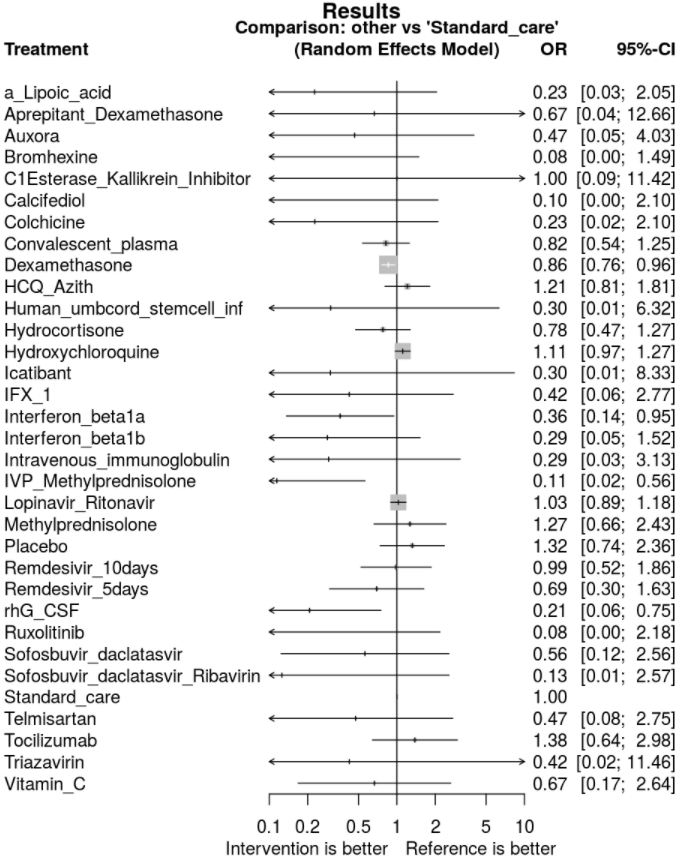


Figure 7 Screenshot of forest plot result of all connected studies (excluding the studies with missing outcomes) as of 19^th^ October 2020 as displayed on the front page

## Detailed data analysis

Above, the results from the random effects NMA model are presented; however, if users wish to compare the NMA results from the fixed effect model, Bayesian analysis, or consider other outcomes, they can do so through the functionalities on the ‘detailed data analysis’ page (Figure 8). A complete user guide on the regular app is provided on the ‘User Guides’ page along with our training videos given as part of Cochrane training webinar series where users can learn more about how to use the detailed data analysis functionalities.


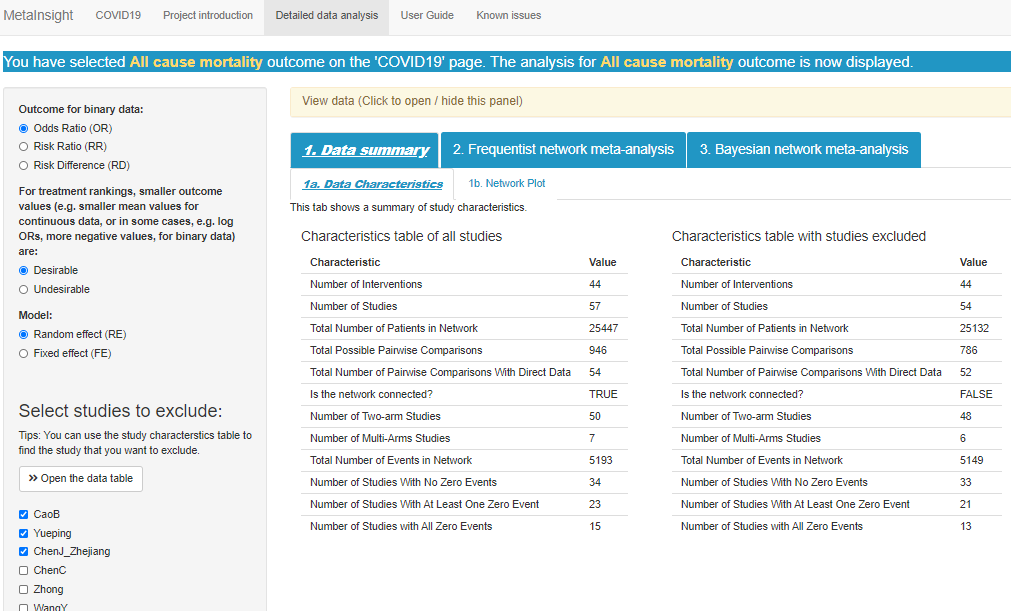


Figure 8 Screenshot of detailed data analysis page
