# Supplementary material for: Feasibility study for interactive reporting of network meta-analysis: experiences from the development of the MetaInsight COVID-19 app for stakeholder exploration, re-analysis and sensitivity analysis from living systematic reviews
Source: BMC Med Res Methodol. 2022 Jan 22;22:26. doi: 10.1186/s12874-022-01507-x (PMC8783587; doi:10.1186/s12874-022-01507-x)
Supplement: Supplementary file 2 — Additional file 2. NMA conducted with CINeMA.docx – NMA conducted with CINeMA – A report of conducting the same NMA using the online web tool CINeMA. [file 12874_2022_1507_MOESM2_ESM.docx]

**Supplemental Material: NMA CONDUCTED WITH CINEMA**

**Title:** Feasibility study for interactive reporting of network meta-analysis: Experiences from the development of the MetaInsight COVID-19 app for stakeholder exploration, re-analysis and sensitivity analysis from living systematic reviews

**Authors:** Yiqiao Xin^1*^, Clareece R Nevill^2*^, Janion Nevill^3^, Ewan Gray^4^, Nicola J Cooper^2^, Naomi Bradbury^5^, Alex J Sutton^2^.
^*^Joint first authorship

**Affiliations:**

1. NIHR Complex Review Support Unit, Health Technology Assessment and Health Economics (HEHTA), Institute of Health and Wellbeing, University of Glasgow, UK.
2. NIHR Complex Review Support Unit, Department of Health Sciences, University of Leicester, UK.
3. Independent researcher, Tewkesbury, UK.
4. Health Economist, Freelance Health Economics consultant, East Lothian, UK.
5. Zeeman Institute: Systems Biology and Infectious Disease Epidemiology Research (SBIDER), School of Life Sciences, University of Warwick, Coventry, UK.

**Corresponding author contact address:**

Alex J Sutton, Department of Health Sciences, Centre for Medicine, University of Leicester, University Road, Leicester, LE1 7RH. [ajs22@leicester.ac.uk](mailto:ajs22@leicester.ac.uk)

**Aim**

To aid comparison of MetaInsight COVID-19 with other similar applications, the network meta-analysis of the primary network from the data currently reported in MetaInsight COVID-19 (https://crsu.shinyapps.io/metainsightcovid/) (extracted 19^th^ October 2020) was conducted using CINeMA (https://cinema.ispm.unibe.ch/).

**Results**

The first page of CINeMA gives a network plot where node size, node colour, edge width, and edge colour can be set to represent characteristics such as number of participants, studies, or risk of bias (RoB) result. Figure 1 is the network plot from our illustrative data, with edge colour representing ‘Average RoB’, and edge width representing number of studies. Unfortunately, the network was too large for CINeMA to print the treatment labels.


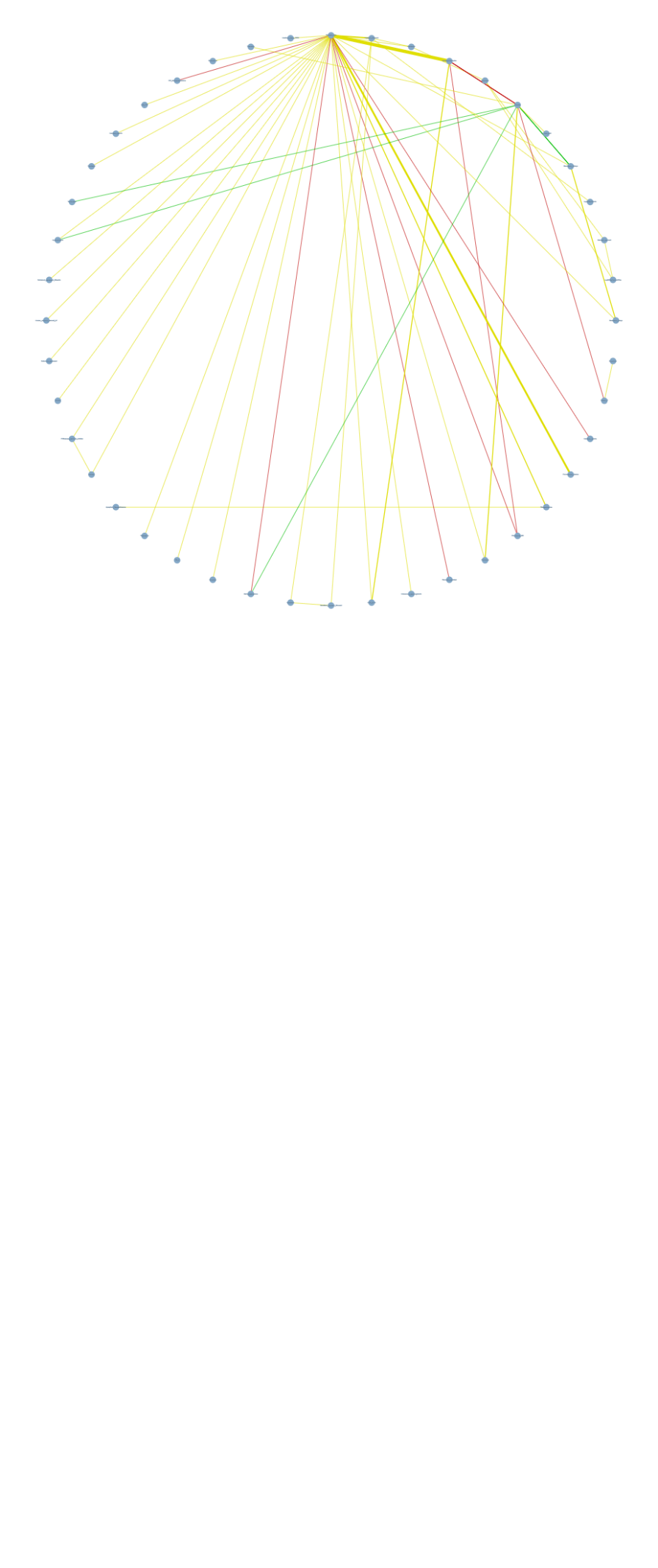


Figure 1: Network plot of COVID-19 treatment studies from CINeMA

To match the summary page of MetaInsight COVID-19, we set the analysis to be from a random-effects model with odds ratios as the outcome. Due to there being 44 treatments in the network, to obtain comprehendible results, we restricted CINeMA to only report results for treatments that had at least three studies with direct evidence, giving 8 treatments and 28 comparisons.

The second page on CINeMA presented the RoB contributions for each treatment comparison – the results for our selection of treatment comparisons, using the same data in MetaInsight COVID-19, is in Figure 2.


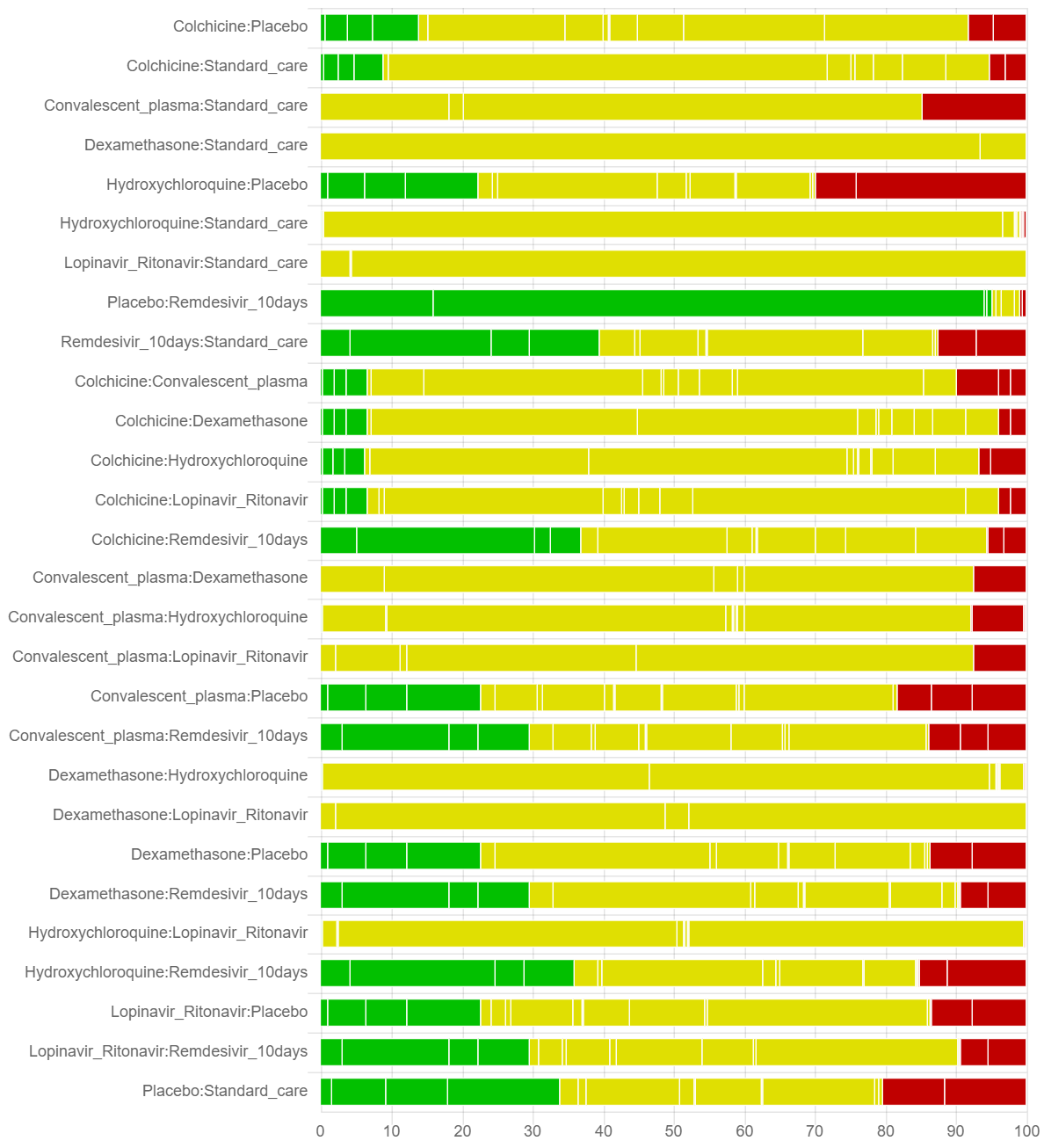


Figure 2: Risk of Bias Contributions table of studies in NMA for COVID-19 treatments, produced by CINeMA, focusing on treatment comparisons that had three or more trials with direct evidence

The ‘reporting bias’ and ‘indirectness’ parts of CINeMA were not run due to lack of necessary data/assessments. For ‘reporting bias’, the level of judgement for reporting bias had to be selected for each treatment comparison (low risk, some concerns, or high risk), informed by completeness of search, considerations related to the field, and statistical methods undertaken. For ‘indirectness’, the study-level indirectness is needed (recorded as low, moderate, or high) which refers to deviations between the data and the targeted research question.
Furthermore, ‘imprecision’, ‘heterogeneity’ and ‘incoherence’ sections were not run as they needed clinical knowledge to define clinically important effect sizes.
